# *Trichoderma harzianum* prevents red kidney bean root rot by increasing plant antioxidant enzyme activity and regulating the rhizosphere microbial community

**DOI:** 10.3389/fmicb.2024.1348680

**Published:** 2024-03-20

**Authors:** Zhifen Guo, Jiaxing Zhang, Zhibin Liu, Yu Li, Meng Li, Qiuxia Meng, Zhiping Yang, Yuan Luo, Qiang Zhang, Min Yan

**Affiliations:** ^1^College of Resources and Environment, Shanxi Agricultural University, Taiyuan, China; ^2^Key Laboratory for Soil Environment and Nutrient Resources, Taiyuan, Shanxi, China; ^3^Institute of Eco-Environment and Industrial Technology, Shanxi Agricultural University, Taiyuan, China; ^4^Shanxi Agricultural University, Taiyuan, China

**Keywords:** red kidney bean root rot, *Trichoderma harzianum*, antioxidant enzyme, rhizosphere microbial community, co-occurrence network

## Abstract

Root rot is one of the main reasons for yield losses of red kidney bean (*Phaseolus vulgaris*) production. Pre-inoculation with *Trichoderma harzianum* can effectively lower the incidence of red kidney bean root rot. In this study, four treatments including CK (control), Fu13 (*Fusarium oxysporum*), T891 (*T. harzianum*) and T891 + Fu13 (*T. harzianum* + *F. oxysporum*) were arranged in a pot experiment to investigate how T891 affected the incidence and severity of root rot, plant growth, and changes of defense enzyme activity in red kidney bean plants. Community composition and diversity of the rhizosphere microbiota was evaluated through high-throughput sequencing, and co-occurrence network was analyzed. The results showed that when compared to the Fu13 treatment, pre-inoculation with T891 reduced the incidence and severity of red kidney bean root rot by 40.62 and 68.03% (*p* < 0.05), increased the root length, shoot length, total dry biomass by 48.63, 97.72, 122.17%. Upregulated activity of super-oxide dismutase (SOD), peroxidase (POD), catalase (CAT) by 7.32, 38.48, 98.31% (*p* < 0.05), and reduced malondialdehyde (MDA) by 23.70% (*p* < 0.05), respectively. Microbiological analyses also showed that *F. oxysporum* reduced alpha diversity resulting in alteration the composition of the rhizosphere microbial community in red kidney bean. T891 significantly reduced abundance of *F. oxysporum*, allowing the enrichment of potentially beneficial bacteria *Porphyrobacter* (ASV 46), *Lysobacter* (ASV 85), *Microbacteriaceae* (ASV 105), and *Gemmatimonas* (ASV 107), resulting in a more stable structure of the microbial network. The results of random forest analysis further revealed that ASV 46 (*Porphyrobacter*) was the primary influencing factor for the incidence of root rot after inoculation with T891, while ASV 85 (*Lysobacter*) was the primary influencing factor for the biomass of red kidney bean. In conclusion, *T. harzianum* promotes the growth of red kidney bean and inhibits root rot by improving plant antioxidant enzyme activity and regulating the rhizosphere microbial community.

## 1 Introduction

Red kidney bean (*Phaseolus vulgaris*) belongs to the *Phaceolus* genus of the Leguminosae family, which has high nutritional value (Koide et al., 1997). Red kidney bean was introduced to Kelan County of Shanxi Province from Italy in 1992, and its sowing area and exports had ranked the first in China since 2017 (Hu et al., 2017). During the past decade, the incidence of root rot in red kidney bean continues to grow due to severe successive cropping. According to the previous research, *Fusarium oxysporum* is the primary causative pathogen of red kidney bean root rot across Shanxi Province (Zhai, 2020), which inhibits the growth of red kidney bean by infecting the rhizomes and roots, leading to the rot of main root and eventually inhibition of height growth yellowing and wilting of the leaves. The annual yield loss caused by root rot in Shanxi ranges from 30 to 80%, resulting in serious economic losses in red kidney bean plantation (Liu et al., 2017).

At present, the main countermeasures to control red kidney bean root rot is still chemical agents. For example, the inhibition rate of seed coating agent containing 11% spermaceti-collo-pyrimidinium and 27% phenyl ether-collo-thiazole suspension against red kidney bean root rot is up to 80.04%, and the inhibition rate of 35% Dockford’s seed coating is 37.23% (Ren et al., 2019). Despite the fact that chemical fungicides may act as an effective tool for managing plant diseases, additional research has revealed that they might also interfere with soil microbiology, not to mention the environmental pollution and the threat to human health they pose (Fliessbach and Mader, 2004; Roth et al., 2020). Therefore, biological control is generating more and more research interest as an substitute for chemical fungicides due to its greenness, safety and efficiency in recent years.

*Trichoderma* is currently one of the most extensively applied biological inoculants for plant disease control. It has been reported that at minimum 60% of registered bio-pesticides globally contain at least one strain of *Trichoderma* (Mukherjee et al., 2013; Singh et al., 2018). This is mainly due to the antagonistic effect of *Trichoderma* on a wide variety of diseases, the promotion of plant growth, the enhancement of plant antioxidant enzyme activity, and the regulation of soil ecological balance (Lorito et al., 2010). Among the *Trichoderma* biologics currently available on the market, *Trichoderma harzianum* products account for the largest proportion and shows good effect (Fraceto et al., 2018; Zin and Badaluddin, 2020). *T. harzianum* Th62 exhibited noteworthy antagonistic activity against 5 soil-borne pathogens, including *F. oxysporum*, *Sclerotinia sclerotiorum*, *Alternaria alternata*, *Cytospora chrysosperma*, and *Rhizoctonia solani*, as reported by Wang et al. (2021). In chili pepper plants infected with *Colletotrichum truncatum*, *T. harzianum* improved activity of superoxide dismutase (SOD), catalase (CAT), and others, reducing the symptoms inflicted by the pathogenic agent and shielding the plants from the disease (Yadav et al., 2021). *T. harzianum* CCTCCRW0024 changes the rhizospheric microbiome of maize and confers disease-resistance against *Fusarium graminearum* (Saravanakumar et al., 2017). However, the biological control of root rot in red kidney bean using *T. harzianum* has not been reported. Little remains of the effect and mechanism of *Trichoderma* against red kidney bean root rot.

Plant health and the prevention of plant diseases depend heavily on the stability and balance that exists between the microbial populations in the rhizosphere and the plants themselves (Gao et al., 2023). For instance, Shahid and Khan (2019) found that *T. viride* resulted in a rapid increase in soil microbial populations but a decrease in the number of pathogenic bacteria, thus effectively prevented root rot in mung bean when infected by *Macrophomina phaseolina*. *T. harzianum* significantly increases the abundance of *Pseudomonas*, *Arthrobacter* and *Flavobacterium*, with no obvious effect on fungal abundance (Zhang et al., 2019). Thus, the soil microorganisms were changed from “fungal type” to “bacterial type,” which is regarded as healthy soil microbiota and the occurrence of crop pests and diseases are reduced. Recognizing the microbial community’s composing in the rhizospheric soil and how it respondes to biological control is key to controlling root rot. However, little literature implicated the effects of inoculation with *T. harzianum* on the rhizosphere microbial community of red kidney bean. Currently, high-throughput sequencing technologies are routinely employed to investigate how biocontrol drugs affect microbial ecosystems. In our previous work, T891, a *T. harzianum* strain obtained from the rhizospheric soil of chili peppers collected in Xinzhou City had shown 74.50% antagonistic effect against red kidney bean root rot caused by *F. oxysporum in vitro*. However its biocontrol effect needs to be future validated, and its action mechanism needs to be unveiled.

Therefore, to determine the biocontrol efficacy of *T. harzianum* T891 pre-inoculation on root rot and the growth promotion effect in red kidney bean plant, and to explore the mechanisms of its regulatory actions, a pot experiment was performed to inspect the effects of T891 inoculation on the incidence of red kidney bean root rot and alterations in the activities of antioxidant enzymes in leaf. High-throughput sequencing was also applied to elucidate the dynamics of rhizosphere microbial community structure. The results of this work may offer some theoretical insights for the use of *T. harzianum* as an alternative fungicide or its application in integrated pathogen management.

## 2 Materials and methods

### 2.1 Materials

*Trichoderma harzianum* T891, *F. oxysporum* Fu13 were isolated and preserved in the Institute of Eco-environment Industrial Technology, Shanxi Agricultural University, and T891 was prepared as spore powder by Shandong Chunong Bio-technology Co. at a concentration of 8 × 10^9^ CFU/g. *F. oxysporum* cakes were cultured in potato dextrose broth (PDB) for 4–5 d to reach the concentration of 1 × 10^7^ CFU/mL for 4°C.

The seeds of red kidney bean (cv. ‘British Red’) were provided by the Agricultural Genetic Resources Research Centre, Shanxi Agricultural University. The soil used was the topsoil collected at the Dongyang Experimental Base of Shanxi Agricultural University (113°76′12″E, 39°74′89″N). The physio-chemical parameters of the soil were: total nitrogen 0.64 g/kg, organic matter 6.60 g/kg, available phosphorus 8.30 mg/kg, available potassium 114.93 mg/kg.

### 2.2 Experimental design

The experiment was conducted from April to May 2023 in the Dongyang Experimental Base of Shanxi Agricultural University (112°34′21″E, 37°46′36″N). Intact red kidney beans were soaked in warm water for about 3 h and sown in seedling trays. Ten days after sowing, 128 red kidney bean seedlings (4 replicates per treatment, 8 plants per replicate) with consistent morphology were transplanted into pots (15 cm × 15 cm) that contained 1.5 kg soil. Four treatments were arranged as control (CK), *T. harzianum* (T891), *F. oxysporum* (Fu13), and *T. harzianum* + *F. oxysporum* (T891 + Fu13). *T. harzianum* inoculant was prepared as a 2 × 10^7^ spore/mL suspension in distilled water and irrigated at 80 mL/pot 3 d after seedling transplantation, and 4 d later *F. oxysporum* suspension at 1 × 10^7^ CFU/mL (50 mL/pot) was irrigated after using the root wounding method (Zhou et al., 2023). The control group used the same amount of distilled water instead when applying suspensions of *T. harzianum* and *F. oxysporum.*

### 2.3 Evaluation of disease index and growth characteristics of red kidney bean

Plant disease incidence was calculated 28 d after inoculation with the pathogen according to Saltos et al. (2021), and disease severity was assessed and control efficacy was calculated according to Guo et al. (2016). The disease severity in each plant was ranked from 0 to 5 based on the following criteria: Grade 0: no rot spots on surface of root and rhizome. Grade 1: sporadic but not contiguous rot spots on root and rhizome, and no spots on fibrous root. Grade 2: the area of rot spots accounts for less than 1/4 of the total surface area of root and rhizome, and fibrous roots begin to develop spots slightly. Grade 3: the area of rot spots covers1/4 to 1/2 of the total surface area of root and rhizome, and a reduction in fibrous roots. Grade 4: the area of rot spots accounts for more than 1/2 of the total surface area of root and rhizome, with contiguous spots on fibrous roots, and some of the fibrous roots fall off. Grade 5: root and rhizome are covered with rot spots, with fibrous roots fall off, and the tap root turns black and short. Plant growth indicators (root length, shoot length, root dry biomass, and shoot dry biomass) were measured as depicted by Shen et al. (2019).

Disease index and relative prevention effect were calculated using the following equations:


$$\mathrm{Disease}\;\mathrm{incidence}\;\left(\%\right)=\;\frac{\left(\mathrm{count}\;\mathrm{of}\;\mathrm{diseased}\;\mathrm{plants}\right)}{\left(\mathrm{total}\;\mathrm{count}\;\mathrm{of}\;\mathrm{tested}\;\mathrm{plants}\right)}\times 100$$

$$\begin{array}{l} \mathrm{Disease}\;\mathrm{index}\;\left(\%\right)=\\ \frac{\sum \left(\left(\mathrm{count}\;\mathrm{of}\;\mathrm{diseased}\;\mathrm{plants}\;\mathrm{at}\;a\;\mathrm{specific}\;\mathrm{stage}\right)\times \left(\mathrm{relative}\;\mathrm{value}\right)\right)}{\left(\mathrm{total}\;\mathrm{count}\;\mathrm{of}\;\mathrm{tested}\;\mathrm{plants}\right)\times \left(\mathrm{highest}\;\mathrm{incidence}\;\mathrm{of}\;\mathrm{disease}\right)}\times 100 \end{array}$$

$$\begin{array}{l} \mathrm{Relative}\;\mathrm{prevention}\;\mathrm{effect}\;\left(\%\right)=\\ \;\frac{\left(\mathrm{disease}\;\mathrm{index}\;\mathrm{of}\;\mathrm{control}\;\mathrm{group}\right)-\;\left(\mathrm{disease}\;\mathrm{index}\;\mathrm{of}\;\mathrm{treated}\;\mathrm{group}\right)}{\left(\mathrm{disease}\;\mathrm{index}\;\mathrm{of}\;\mathrm{control}\;\mathrm{group}\right)}\times 100 \end{array}$$

### 2.4 Enzyme activities leaves

The third fully expanded leaf was collected, washed and immediately placed in liquid nitrogen for freezing at the time of harvest, and then carried to the lab and stored at −80°C. The nitro blue tetrazolium method was used to test the activity of SOD. The guaiacol colorimetric approach was utilized to determine the activity of peroxidase (POD). Potassium permanganate titration was used to evaluate the activity of CAT. Utilizing chromatometry and the thiobarbituric acid technique, the malondialdehyde (MDA) content was analyzed (Li et al., 2021). Four replicates were set for each treatment.

### 2.5 DNA extraction

Four weeks after transplantation, the procedures described by Bulgarelli et al. (2012) were followed to collect the rhizosphere soil samples. The loosely attached soil was removed by shaking and then around 1 mm of soil at the surface of roots was gathered using sterile brush. Following the removal of plant remnants, the soil samples were stored at −80°C. Four samples for each treatment were collected as replicates.

Total genomic DNA samples of soil were extracted using the OMEGA Soil DNA Kit (M5635-02) (Omega Bio-Tek, Norcross, GA, USA), as directed by the manufacturer, and stored at −20°C until further analysis. Using an agarose gel electrophoresis and a NanoDrop NC2000 spectrophotometer (Thermo Fisher Scientific, Waltham, MA, USA), the quantity and purity of extracted DNAs were tested.

### 2.6 16S rRNA gene and its amplicon sequencing

During PCR amplification of the V3-V4 region of the 16S rRNA gene in bacteria, forward primer 338F (5′-ACTCCTACGGGAGG CAGCA-3′) and the reverse primer 806R (5′-GGACTACHVGGGTWT CTAAT-3′) were used. When amplifying the fungal ITS1 region by PCR, the forward primer ITS1F (5′-CTTGGTCATTTAGAGG AAGTAA-3′) and the reverse primer ITS2R (5′-GCTGCGTTCTTC ATCGATGC-3′) were selected. Before multiplex sequencing, sample-specific 7-bp barcodes were added to the primers, and then sequencing was performed according to the protocol. The PCR components consisted 5 μL of buffer (5×), 0.25 μL of Fast Pfu DNA polymerase (5 U/μl), 2 μL (2.5 mM) of dNTPs, 1 μL (10 μM) of each forward and reverse primer, 1 μL of DNA template, and 14.75 μL of ddH_2_O (Sun et al., 2022). PCR reactions were performed as follows: 98°C for 5 min, and then there are 25 cycles for bacteria and 28 cycles for fungus, which consisting of denaturation at 98°C for 30 s, annealing at 53°C for 30 s for bacteria, annealing at 55°C for 30 s for fungi, and extension at 72°C for 45 s, with a final extension of 5 min at 72°C. The obtained PCR amplicons were purified with Vazyme VAHTSTM DNA Clean Beads (Vazyme, Nanjing, China) and subsequently quantified via the Quant-iT PicoGreen dsDNA Assay Kit (Invitrogen, Carlsbad, CA, USA). After the individual quantification procedure, amplicons were pooled in equal amounts, and pair-end 2 × 250 bp sequencing was carried out at an Illlumina NovaSeq platform using NovaSeq 6000 SP Reagent Kit (500 cycles) (Yuan et al., 2023).

QIIME2 (2019.4) software was used to call qiime cutadapt trim-pairs, so as to remove the primer fragments and cast away the sequences of the unmatched primers. Subsequently, qiime dada2 noise-paired named DADA2 was used to perform quality control, denoising, splicing, and chimera removal, which was equivalent to 100% similar clustering (Callahan et al., 2016). Statistical analysis was then performed to produce the amplicon sequence variants (ASVs) required for species assignment (Li et al., 2023). Furthermore, after methodically eliminated the sequences annotated as mitochondria and chloroplasts in each sample, subsampling was done to guarantee that the sample sequence numbers were consistent (Zhang et al., 2023a).

### 2.7 Microbial network analysis

The Molecular Ecological Network Analysis Pipeline was used for co-occurrence network analysis (Deng et al., 2012). The top 410 ASVs (relative abundance>0.01%, occured in at minimum 80% of all samples) were retained for analyses to enhance the dependability of the network construction, and the network was constructed with the same threshold after threshold scanning via an RMT-based method (St = 0.88) to construct the network (Deng et al., 2012). The construction of the network graph was performed at the Gephi platform (Ling et al., 2016). Based on intra-module connectivity (Zi) and inter-module connectivity (Pi) each node’s connectedness was ascertained (Guimerà and Nunes Amaral, 2005). With thresholds of 2.5 and 0.62, respectively, the values of Zi and Pi were used to determine the possible keystone genera (Wu et al., 2022).

### 2.8 Statistical analyses

Sequence data analyses were primarily carried out using the QIIME2 and R software packages (v3.2.0). All the analyses of 16S rDNA and ITS amplicon sequencing data for the microbial community were conducted based on normalized ASVs table. Chao1 richness, Shannon diversity index and Pielou_e evenness were calculated using the ASVs table in QIIME2. Significance was analyzed using one-way ANOVA using SPSS 27.0 software and calculated using Tukey’s method (*p* < 0.05). The analysis of principal coordinate analysis (PCoA) based on Bray-Curtis distance was carried out to observe the structural variation of the rhizosphere soil microbiome across samples. The differences in the rhizosphere soil microbiota among different groups were determined by analysis of the similarity (ANOSIM), and the R package “ggtree” was used to visualize and test linear discriminant analysis effect size (LEfSe) to observe the differentially abundant taxa between treatments. To further identify the ASVs associated with different treatments compared to CK, the log2-fold changes in ASVs (log2FC) was calculated using the negative binomial distribution-based “DESeq2” package in R. ASVs with adjusted *p*-value below 0.05 and |log2FC| above 2 were selected (Gao et al., 2023), and Spearman correlation coefficients were used to evaluate the correlation of bacterial and fungal ASVs with disease incidence and plant biomass. Relative importance analyses of microbial indicators with disease incidence and biomass were performed using a random forest model (R package’ random Forest) (Jin et al., 2017).

## 3 Results

### 3.1 Root rot disease index and growth characteristics of red kidney bean

**Figure 1:**
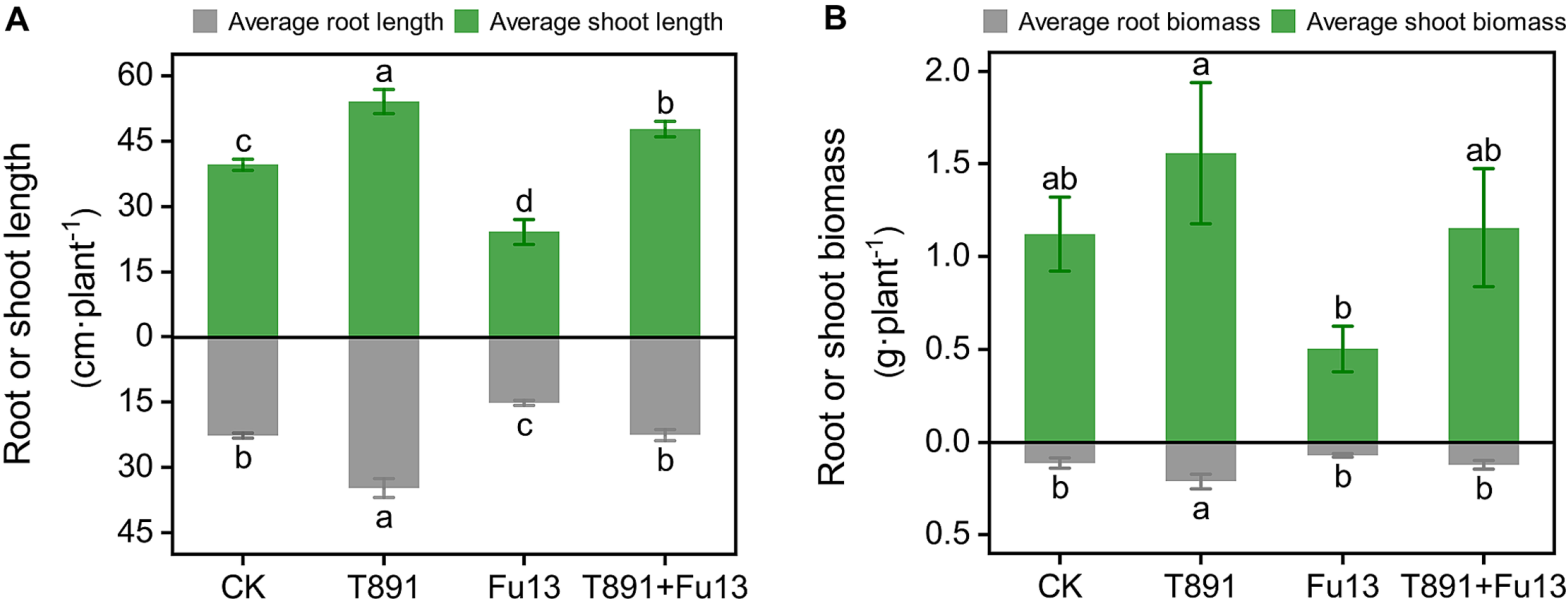
Average root length, stem length **(A)**, and plant biomass **(B)** (mean ± SD) of red kidney bean plants in different treatments. According to Tukey’s test, different letters indicate significant differences between treatments (*p* < 0.05).

After 4 weeks of cultivation in the greenhouse, different disease indices and growth characteristics of red kidney bean were observed in different treatments (Table 1; Figure 1). No significant disease symptoms were found in plants in CK, while significantly increased incidence and disease index were recorded in Fu13 (*p* < 0.05), with an incidence of 100% and a disease index of 76.25%. By contrast, T891 + Fu13 effectively reduced the incidence and disease index of root rot as compared to Fu13 (*p* < 0.05), showing a relative preventive effect of 67.91%. T891 alone significantly improved the growth of red kidney bean plants (*p* < 0.05), and the root length, stem length, and total biomass were increased by 53.19, 36.49, and 46.94%, respectively when compared to CK. Whereas Fu13 alone significantly reduced the growth of red kidney beans plants (*p* < 0.05), and the root length, stem length, and total biomass were decreased by 33.24, 39.02, and 48.57%, respectively. In addition, T891 + Fu13 significantly promoted the root length, stem length and total biomass of red kidney bean plants by 48.63, 97.72 and 122.17%, respectively as compared with Fu13 treatment (*p* < 0.05). It was also interesting to see that the stem length of T891 + Fu13 was increased by 20.58% compared to CK treatment (*p* < 0.05).

**Table 1:** Disease index of red kidney bean root rot under different treatments.

Treatment	Disease incidence (%)	Disease index (%)	Relative prevention effect (%)
CK	0.00 ± 0.00c	0.00 ± 0.00c	–
Fu13	100.00 ± 0.00a	76.25 ± 4.33a	–
T891 + Fu13	59.38 ± 6.25b	24.38 ± 2.39b	67.91 ± 0.04

Data were presented as mean ± SD deviation. According to Tukey’s test, values with different lowercase letters in the same columns indicate significant differences (*p* < 0.05).

### 3.2 Antioxidative enzyme activity and MDA content in red kidney bean leaves

**Figure 2:**
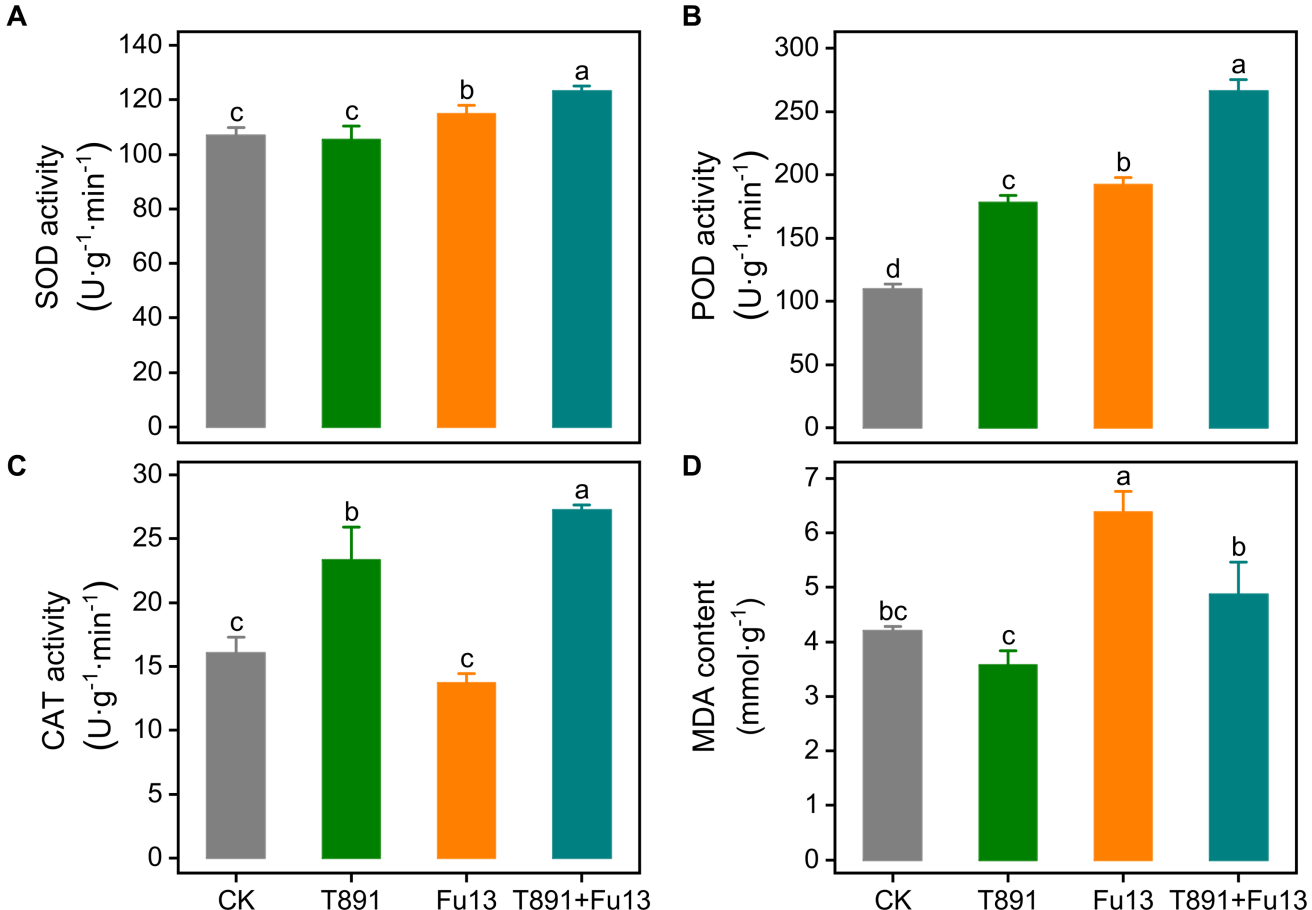
Effect of *T. harzianum* application on activity of SOD **(A)**, POD **(B)**, CAT **(C)** and content of MDA **(D)** in red kidney bean leaves (mean ± SD). According to Tukey’s test, different letters indicate significant differences between treatments (*p* < 0.05).

After 4 weeks of growth, the activity of antioxidative enzymes in red kidney bean leaves was significantly different in the four treatment groups (Figure 2). No significant change was found in SOD activity of red kidney bean treated with T891 as compared to CK (*p* > 0.05), while SOD activity underwent a significant increase of 7.37% (*p* < 0.05) in the Fu13 treatment and was raised to an even higher level in the T891 + Fu13 treatment, which was 15.23% higher than that of CK (*p* < 0.05). Furthermore, SOD activity in T891 + Fu13 was increased by 7.32% compared to that in Fu13 (*p* < 0.05). The highest level of POD activity was recorded in the T891 + Fu13 treatment and increased by 142.84% as compared to CK, followed by the treatments of Fu13 and T891, which were increased by 75.37 and 62.82%, respectively, and the lowest level was found in CK. Notably, POD activity in T891 + Fu13 was increased by 38.48% compared with Fu13 (*p* < 0.05). Significant differences were identified among four groups regarding the POD activity (*p* < 0.05). By contrast, the infection of Fu13 did not caused changes in CAT activity (*p* > 0.05), but the inoculation of T891 and the combined application of T891 and Fu13 resulted in significant increases by 45.55 and 69.71%, respectively, when compared to CK (*p* < 0.05). Also, CAT activity in T891 + Fu13 was significantly increased by 98.31% when compared with Fu13 (*p* < 0.05). The MDA content was abated by 14.94% after the inoculation of T891 but was raised by 51.96% after treating with Fu13 (*p* < 0.05). Meanwhile, the pre-inoculation of T891 reversed the increase of MDA content and reduced it to a level similar to that of control (*p* > 0.05). In T891 + Fu13 the MDA content was remarkably abated by 23.70% compared to Fu13 (*p* < 0.05).

### 3.3 Diversity and structure of the microbial communities

#### 3.3.1 Alpha diversity analysis

**Figure 3:**
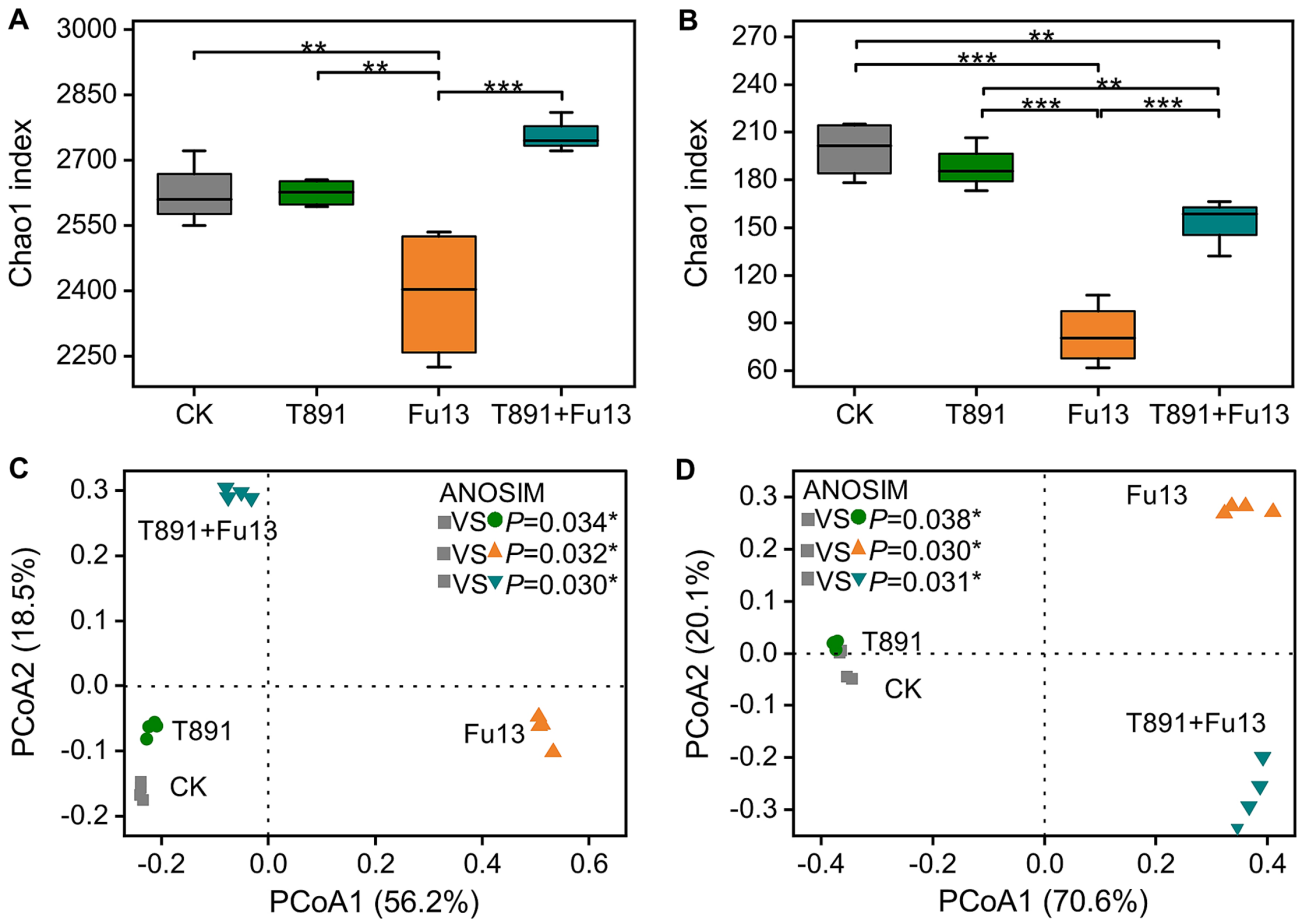
Bacteria **(A)** and fungi **(B)** richness (Chao1) in different treatments, principal coordinate analysis (PCoA) of bacterial **(C)** and fungal **(D)** community composition in all soil samples based on Bray-Curtis distance metrics. Box plots show the first and third quartiles with horizontal bars at the median and whisker lines show the range of outliers, not exceeding 1.5 times the interquartile range. *p < 0.05, **p < 0.01, ***p < 0.001 according to Tukey’s test.

The values of alpha diversity are shown in Figures 3A,B and Supplementary Figure S1. The application of T891 did not have significant effect on alpha diversity of bacterial and fungal compared to CK (*p* > 0.05), while the pathogen Fu13 significantly decreased the alpha diversity indices of bacterial and fungal communities in the rhizospheric soil of red kidney bean (*p* < 0.01). In the T891 + Fu13 treatment the deceased of alpha diversity of bacterial was reversed, which significantly increased when compared to the Fu13 treatment. Whereas in the T891 + Fu13 treatment the decrease of fungal community richness (Chao1) was also reversed as compared to CK (*p* < 0.01) although still lowered than those of the T891 treatment or CK, and no significant differences were identified in diversity (Shannon) and evenness (Pielou_e) indices of bacterial and fungal (*p* > 0.05).

#### 3.3.2 Principal coordinate analysis

Principal coordinate analysis (PCoA) and ANOSIM analyses revealed significant differences in the composition of bacterial (*p <* 0.05, Figure 3C) and fungal (*p <* 0.05, Figure 3D) communities between the four groups. The 1st and 2nd axes indicate the two selected principal component axes and the percentages represents the values of the degree of interpretation of the first and second principal components to the differences in the sample structure, which explain 56.2 and 18.5% of the variations, respectively, and 74.7% of the total variations for the bacterial community. Regarding the fungal community, the two selected principal components explain 70.6 and 20.1% of the variations and 90.7% of the total variations.

#### 3.3.3 Linear discriminant analysis effect size

LEfSe analyses revealed that when the LDA threshold was set to 4.5, the number of bacterial microbiota (Supplementary Figure S2A) was 5 (g_Streptomyces, f_Streptomycetaceae, o_Streptomycetales, c_Actinobacteria, p_Actinobacteriota) in CK, 2 (o_Streptosporangiales, f_Rhodanobacteraceae) in T891, 13 (g_Sphingobacterium, f_Sphingobacteriaceae, o_Sphingobacteriales, c_Bacteroidia, p_Bacteroidota, g_Rheinheimera, f_Alteromonadaceae, o_Enterobacterales, g_Pseudomonas, f_Pseudomonadaceae, o_Pseudomonadales, c_Gammaproteobacteria, p_Proteobacteria) in Fu13, and 3 (g_unclassified_Micrococcaceae, f_Micrococcaceae, o_Micrococcales) in T891 + Fu13, respectively. The number of fungal community (Supplementary Figure S2B) was 3 (g_Chaetomidium, f_Chaetomiaceae, o_Sordariales) in CK, 4 (g_Geomyces, g_Trichoderma, f_Hypocreaceae, p_Ascomycota) in T891, 4 (g_Fusarium, f_Nectriaceae, o_Hypocreaceae, c_Sordariomycetes) in Fu13 and 5 (g_Pseudogymnoascus, f_Pseudeurotiaceae, o_Thelebolales, c_Leotiomycetes, g _Botryotrichum) in T891 + Fu13, respectively. The results suggest that the different treatments might have altered the microbiota and these specific taxa could be possible biomarker species for them.

### 3.4 Microbial community composition

**Figure 4:**
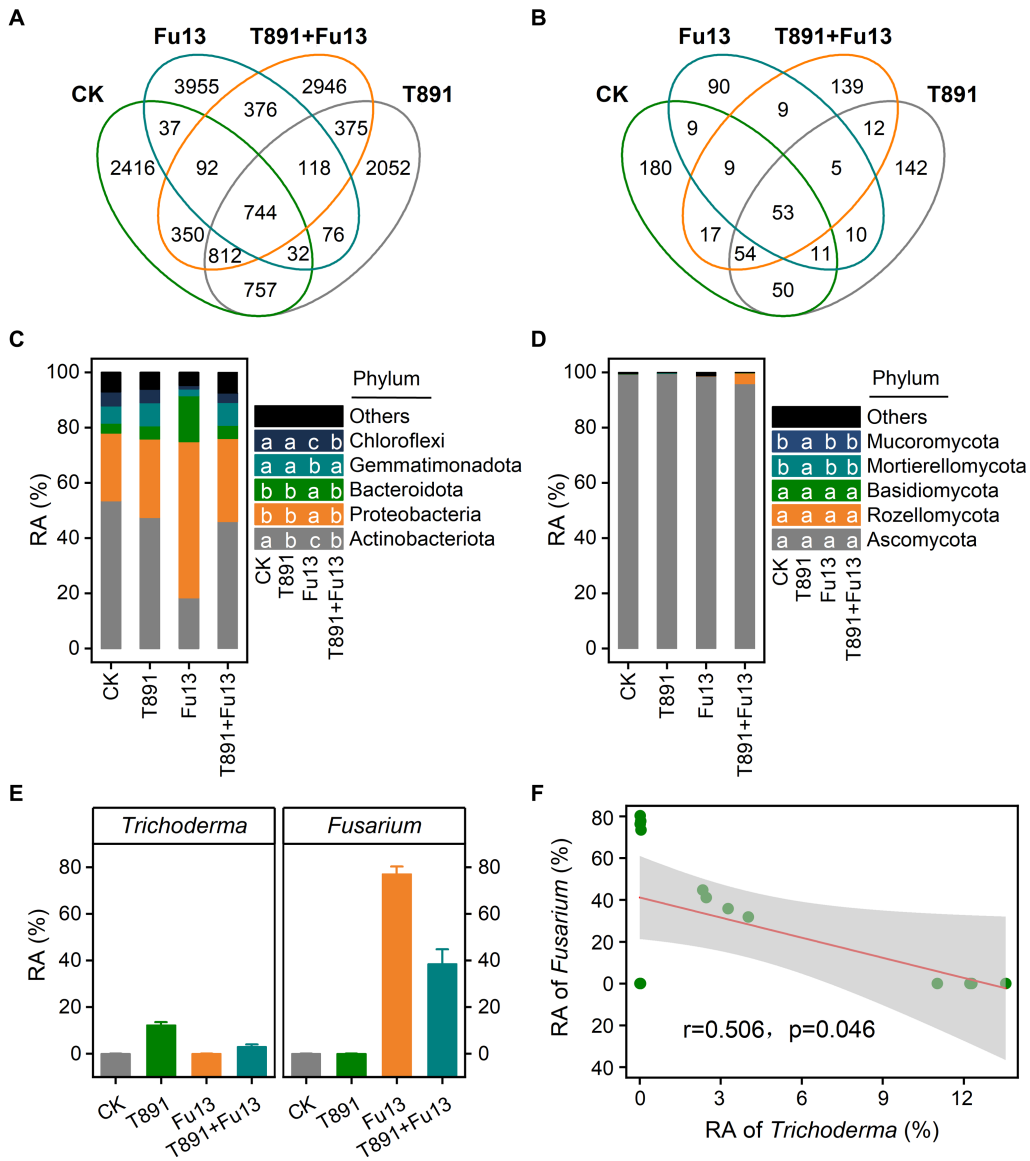
Venn diagram for the number of shared and unique bacterial **(A)** and fungal **(B)** ASVs under different treatments, relative abundance (RA) of bacterial **(C)** and fungal **(D)** communities at the phylum level of rhizosphere soil under different treatments, and relative abundance (mean ± SD) of *Trichoderma* and *Fusarium* in the different treatments **(E)** as well as Spearman correlations between relative abundance of *Trichoderma* and *Fusarium* **(F)**. According to Tukey’s test, different letters indicate significant differences between treatments (*p* < 0.05).

For all samples, 15,138 bacterial ASVs and 792 fungal ASVs were found totally (Figures 4A,B). 5,240, 4,966, 5,430, 5,813 bacterial ASVs and 383, 337, 196, 298 fungal ASVs were identified for CK, T891, Fu13, and T891 + Fu13 samples, respectively. Notably, more unique bacterial ASVs and less unique fungal ASVs were found in the Fu13 treatment relative to other treatments, suggesting that the addition of Fu13 had a distinct stimulatory action on the composition of rhizosphere microbial community in red kidney bean.

The assembly of the top 5 bacterial and fungal phyla in the rhizospheric soil of red kidney bean is shown in Figures 4C,D, respectively. In the bacterial community, T891 reduced the relative abundance of *Actinobacteriota* when compared with CK (*p* < 0.05), while Fu13 lowered the relative abundance of *Actinobacteriota*, *Gemmatimonadota* and *Chloroflexi* and increased the relative abundance of *Proteobacteria* and *Bacteroidota* (*p* < 0.05). Compared to Fu13, T891 + Fu13 increased the relative abundance of *Actinobacteriota*, *Gemmatimonadota*, and *Chloroflexi* when decreased the relative abundance of *Proteobacteria* and *Bacteroidota* (*p* < 0.05). *Ascomycota* was present as the phylum with the highest relative abundance in all groups within the fungal community (*p* < 0.05), accounting for 99.40, 99.62%, 98.70 and 95.54% of the fungal population in CK, T891, Fu13 and T891 + Fu13, respectively. In addition, only *Mucoromycota* and *Mortierellomycota* showed higher relative abundance in the T891 treatment relative to other three groups (*p* < 0.05), whereas no significant difference was observed in the other three groups (*p* > 0.05).

Further analysis of the microbial community showed that *Trichoderma* accounted for 0.01, 12.27, 0.02 and 3.01% of the total fungal community and *Fusarium* accounted for 0.06, 0.06, 77.02 and 38.41% of the total fungal community in the CK, T891, Fu13 and T891 + Fu13 treatments, respectively. The T891 + Fu13 treatment significantly reduced the abundance of *Fusarium* compared to Fu13 (*p* < 0.05), and a significant negative correlation was observed between the relative abundance of *Trichoderma* and *Fusarium* (Figures 4E,F).

### 3.5 Co-occurrence networks of microbial communities

**Figure 5:**
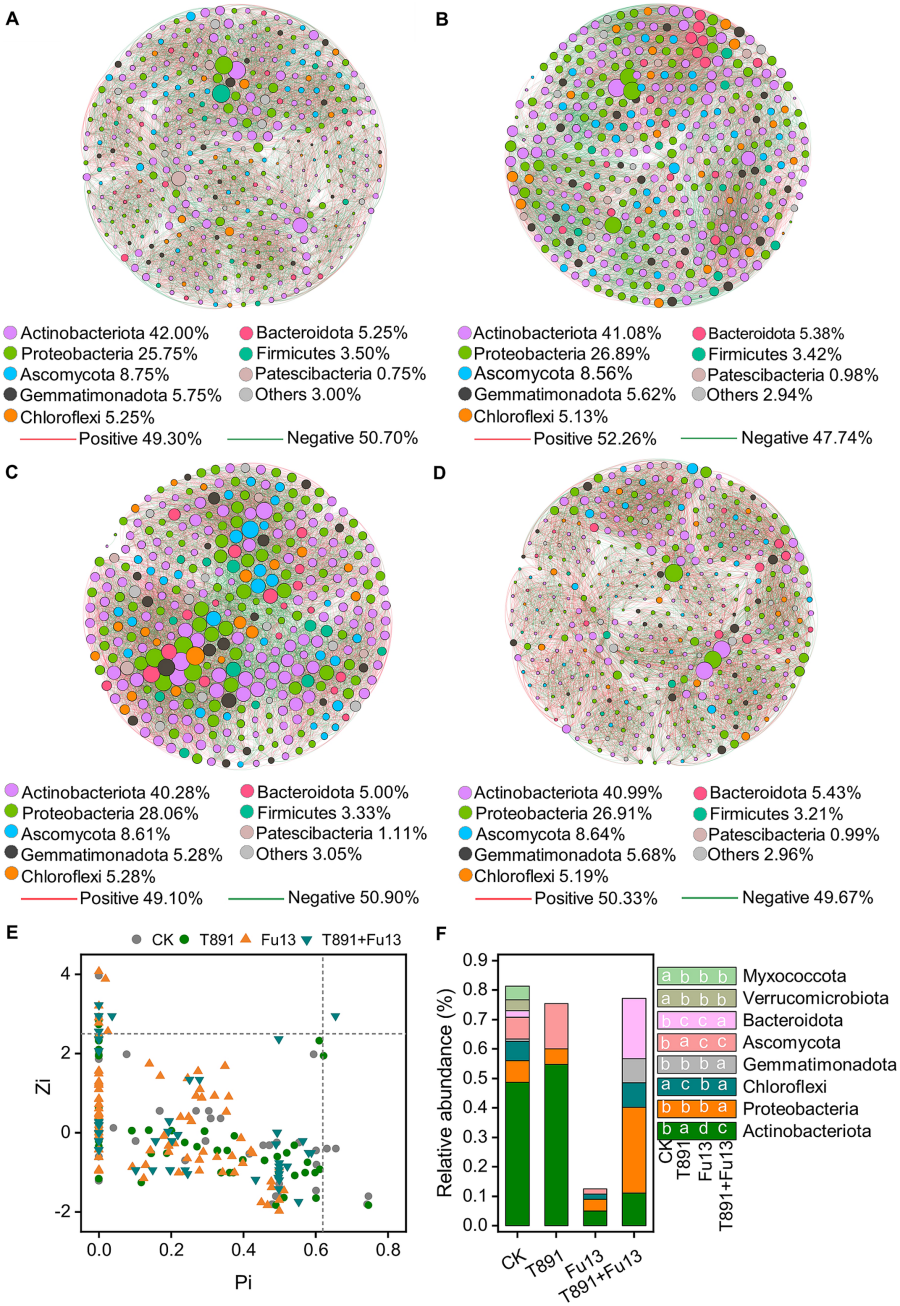
Co-occurrence network of ASVs in CK **(A)**, T891 **(B)**, Fu13 **(C)** and T891 + Fu13 **(D)**, Zi-Pi of the distribution of ASVs based on their topological roles **(E)** and the composition of key ASVs in different treatments **(F)**. The size of each node is proportional to the number of connections (degree). The color of connections between two nodes represents positive (red) or negative (green) correlation. The node colors show various phyla.

The co-occurrence networks of CK, T891, Fu13 and T891 + Fu13 were composed of 400, 409, 360 and 405 nodes, respectively as displayed in Figure 5 and Table 2. Most of the nodes in the four networks belonged mainly to the strains *Actinobacteriota* (CK 42%, T891 41.08%, Fu13 40.28%, T891 + Fu13 40.99%), *Proteobacteria* (CK 25.75%, T891 26.89%, Fu13 28.06%, T891 + Fu13 26.91%) and *Ascomycota* (CK 8.75%, T891 8.56%, Fu13 8.61%, T891 + Fu13 8.64%). A total of 9,629, 9,383, 9,796, and 9,396 co-occurring relationships were inferred from the consistent microbial networks of CK, T891, Fu13, and T891 + Fu13, respectively. CK (49.30%), Fu13 (49.10%) and T891 + Fu13 (50.33%) had fewer positive interactions in the microbial community compared to T891 (52.26%), suggesting that they had more intergeneric competition.

**Table 2:** Topological properties of the rhizosphere microbiome-associated networks of red kidney bean under different treatments.

Network metrics	CK	T891	Fu13	T891 + Fu13
Nodes	400	409	360	405
Edges	9,629	9,383	9,796	9,396
Average degree	48.145	45.883	54.422	46.400
Average path distance	3.096	2.914	2.387	3.150
Average clustering coefficient	0.834	0.832	0.751	0.872
Modularity	0.602	0.643	0.546	0.654

Multiple topology metrics showed different complexity and stability of microbial networks in various groups (Table 2). In terms of average degree, the network of Fu13 is more complex and has higher numbers of connections between nodes. In terms of average path length, the network of T891 + Fu13 had the highest degree of connectivity between nodes, average clustering coefficient and modularity index. This evidenced that when red kidney bean was disturbed by the pathogenic bacterium Fu13, the presence of *Trichoderma* led to a higher degree of connectivity between adjacent nodes of the microbial network, and the nodes were more likely to cluster together to form a tight modular structure, which facilitated the stability of the microbial ecosystem.

The topological characteristics of each node are shown using a Zi-Pi diagram (Figure 5E). Most (96.38%) of the nodes are peripherals since the majority of their links are within the modules, around 2.86% of the nodes are module hubs and 0.70% of the nodes are connectors. The Fu13 treatment has the lowest relative abundance of key nodes (Pi>0.62 or Zi > 2.5) compared to the other three treatments (Figure 5F). Only one ASV network hub was present in the T891 + Fu13 treatment, and these differences in key ASVs similarly confirmed that *Trichoderma* and *Fusarium* significantly changed the structural diversity and composition of rhizosphere soil microbial community in red kidney bean.

### 3.6 Effect of different treatments on potentially beneficial microorganisms

**Figure 6:**
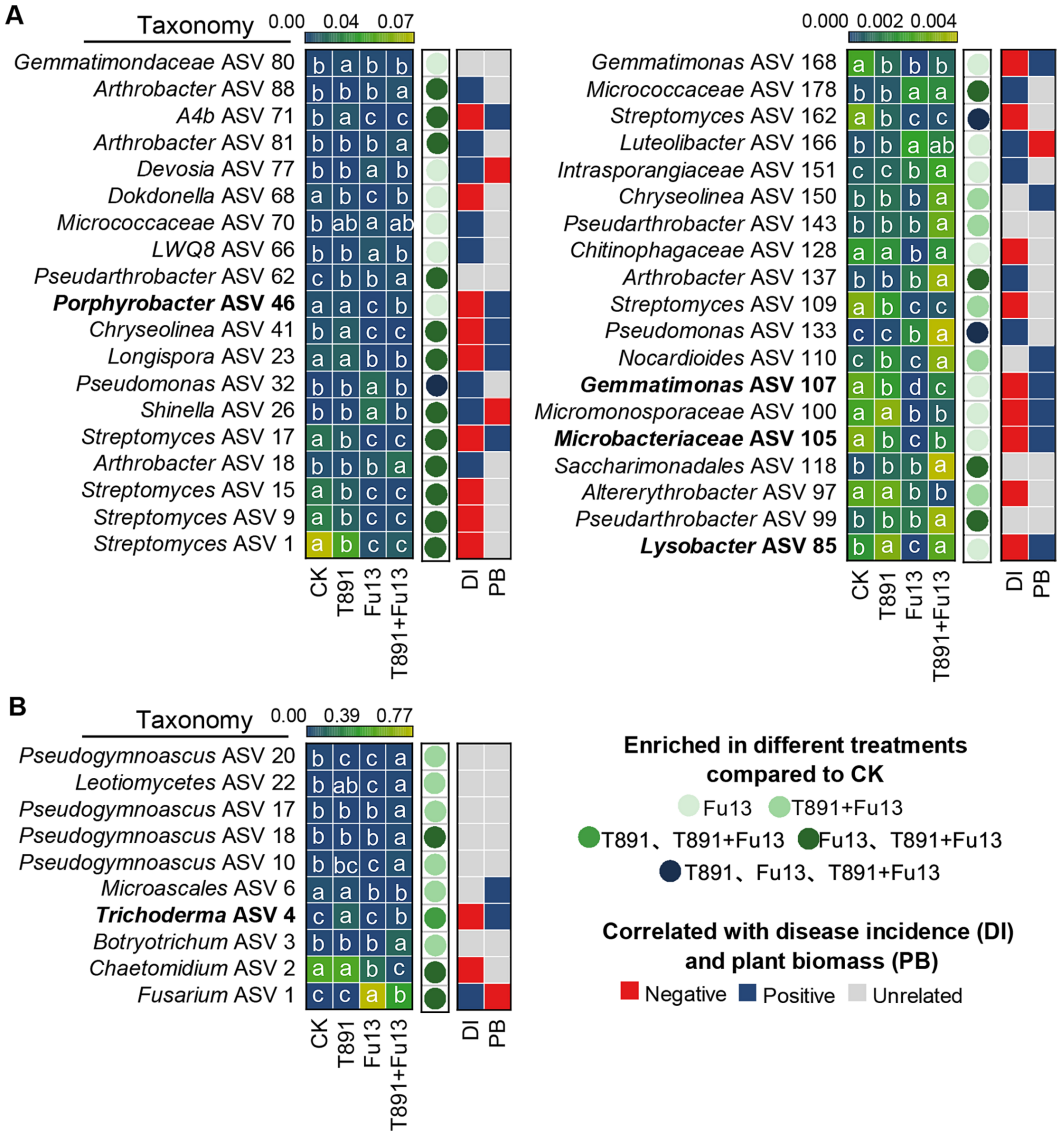
Heat map showing the core bacterial **(A)** and fungal **(B)** microbiota as affected by different treatments and correlated with plant disease incidence (DI) and biomass (PB). Colored circles indicate the differential abundance of ASVs found in the inter-root soils of different treatments, respectively. |log2FC| > 2 and an FDR adjusted *p*-values<0.05 is considered differentially enriched. Colored squares indicate correlations with disease incidence and plant biomass.

After removal of ASVs with a relative abundance below 0.1% in tested soil samples, 174 bacterial ASVs, 70 fungal ASVs were recovered in total, of which 127 bacterial ASVs and 25 fungal ASVs were identified as core ASVs due to their presence in at least 80% of all samples. From the results of the DESeq2 algorithm, it was clear that the different treatments exerted greater influence on the bacterial community than on the fungal one (Figure 6, Supplementary Table S1, |log2FC| > 2, padj<0.05). In the T891, Fu13 and T891 + Fu13 treatments 3, 33 and 24 bacterial ASVs were affected, respectively (Figure 6A). Three of these ASVs were affected in the T891, Fu13 and T891 + Fu13 treatments, and 16 ASVs were affected in both Fu13 and T891 + Fu13 treatments. Spearman correlation analysis revealed that the relative abundance of 10 ASVs was negatively associated with disease incidence or positively associated with red kidney bean biomass, and were therefore considered to be putatively beneficial bacteria. The relative abundance of four of these ASVs was significantly higher in the T891 + Fu13 treatment than in the Fu13 treatment (*p* < 0.05), and were found to belong to *Porphyrobacter* (ASV 46), *Lysobacter* (ASV 85), *Microbacteriaceae* (ASV 105) and *Gemmatimonas* (ASV 107), with 17.75-, 23.94-, 17.15-, and 21.00- fold increases in relative abundance, respectively. In the T891, Fu13 and T891 + Fu13 treatments, 1, 3 and 10 fungal ASVs were affected, respectively (Figure 6B). Among them, 1 ASV was affected in both T891 and T891 + Fu13 treatments, and 3 ASVs were affected in both Fu13 and T891 + Fu13 treatments, Spearman’s correlation analysis revealed that only ASV 4 was negatively associated with disease incidence and positively associated with red kidney bean biomass at the same time, and ASV 4 was identified as *Trichoderma*.

### 3.7 Relative importance analysis of microbial indicators with disease incidence and biomass

**Figure 7:**
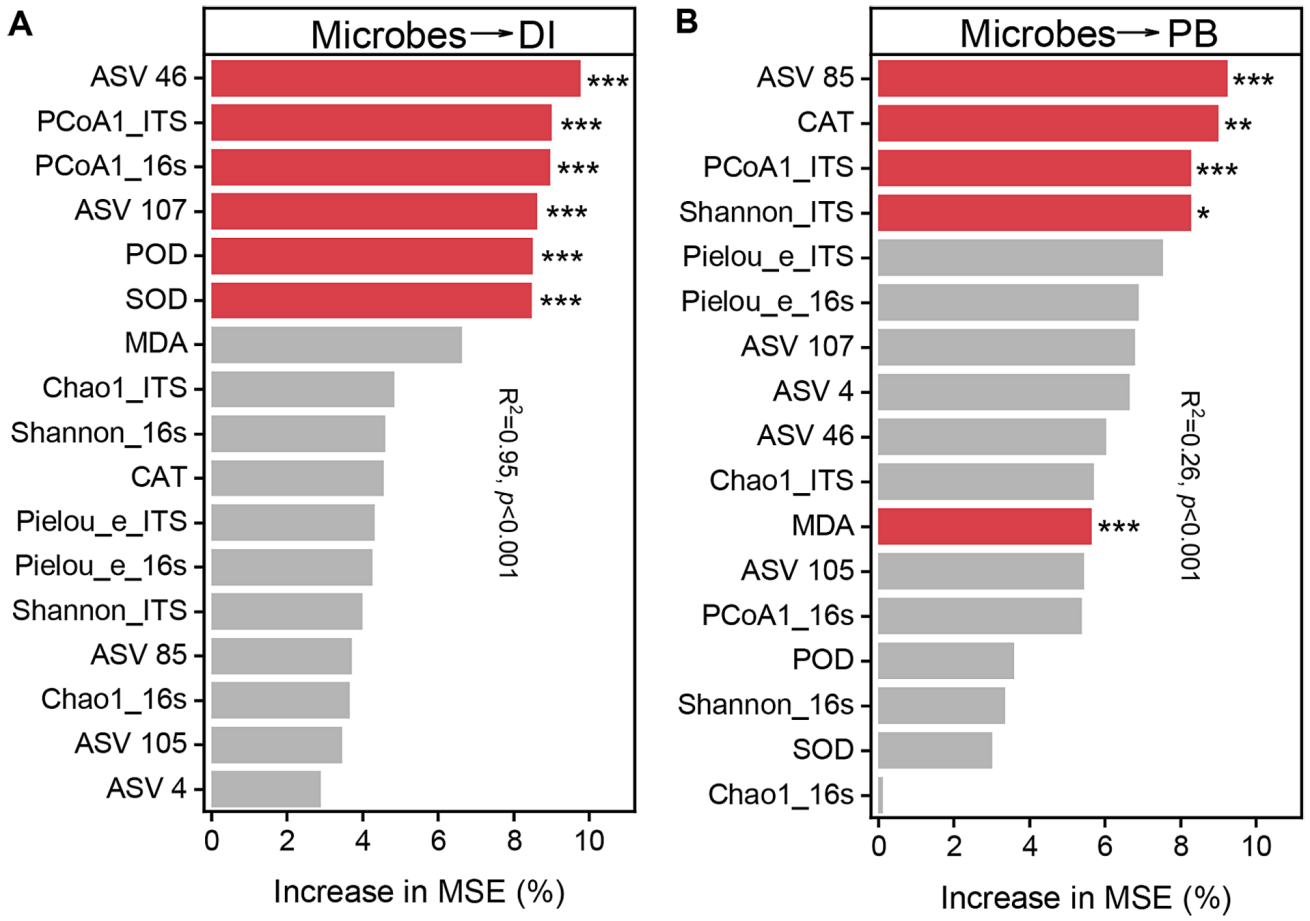
Relative importance of microbiological indicators for disease incidence (DI) **(A)** and biomass (PB) **(B)** (percentage increase in mean square error). The importance of these indicators was estimated using the percentage increase in the mean square error (MSE) of the variables, with higher MSE% values implying greater importance. Significance levels are as follows: *p < 0.05, **p < 0.01, ***p < 0.001.

To further explain the principle influencing factors of disease incidence and biomass, Chao1 richness, Shannon diversity index and Pielou_e evenness, PCoA1, potentially beneficial microorganisms ASV46 (*Porphyrobacter*), ASV 85 (*Lysobacter*), ASV 105 (*Microbacteriaceae*), ASV 107 (*Gemmatimonas*), and ASV 4 (*Trichoder*ma), and the activities of induced systemic resistance (ISR) related enzymes SOD, POD and CAT, and the MDA content were analyzed using Random Forest Model (Figure 7), which showed that ASV 46 (9.74%), PCoA1_ITS (9.00%), PCoA1_16s (8.95%), ASV 107 (8.61%), POD (8.49%) and SOD (8.46%) were the main influencing factors on root rot incidence (*p* < 0.001), and ASV 85 (9.23%), CAT (8.98%), PCoA1_ITS (8.27%), Shannon_ITS (8.26%), and MDA (5.63%) were the main influencing factors on biomass (*p* < 0.05).

## 4 Discussion

### 4.1 *Trichoderma harzianum* T891 reduces red kidney bean root rot and improves plant growth index and antioxidative enzyme activity

*Trichoderma* spp. has been extensively used to harness various soil-borne plant diseases. For example, *T. harzianum* can effectively control root diseases of cucumber, chili, potato and other crops (Ahmed et al., 1999; Yang et al., 2011; Mohamed et al., 2020). Nevertheless, differences in plant species, geographical location and microbial communities may lead to variances in control effects across reports (Li et al., 2020). For instance, Saravanakumar et al. (2017) found that *T. harzianum* reduced the incidence of maize *Fusarium* stalk rot by 86.66% and altered the assembly and structure of the microbiota; Li et al. (2020) found that *T. harzianum* reduced cabbage rhizoctonia by 45.4%, with a disease control effect of 63%, and that the decrease in incidence and severity were correlated to the dramatic drop in the relative abundance of the causal organisms of cabbage rhizoctonia. Our current work showed that the application of T891 reduced the incidence of red kidney bean root rot from 100 to 59.38%, with a relative efficacy of 67.91% (Table 1). Moreover, the rhizospheric soil of the T891 + Fu13 treatment exhibited a greater abundance of *Trichoderma* and a significantly smaller abundance of *Fusarium* (Figure 4E) when compared to the Fu13 treatment, suggesting that the use of *T. harzianum* had a prominent control effect on *F. oxysporum* in the presence of a complex indigenous microbial community. In addition, our experiments revealed that T891 produced the highest biomass of red kidney bean plant (Figure 1), which was 43.47, 207.83 and 38.55% higher than CK, Fu13, and T891 + Fu13, respectively. This coincides with the early reports that suggest *T. harzianum* has an extra benefit of improving plant growth and yield (Zin and Badaluddin, 2020).

The antioxidative enzymes including SOD, POD and CAT are essential for plants to counteract environmental stresses (Kusvuran, 2021), and plant-associated fungi have been reported to generate or secrete a range of compounds that activate plant immunity against pathogen infection (Salwan et al., 2022). Zhang et al. (2011) suggested that POD and Phenylalanine ammonia lyase (PAL) activity was enhanced in maize seeds treated with *Trichoderma* coating and that the plants were conferred resistance against maize *Curvularia* leaf spot. Inoculation of *T. harzianum* spore suspension on cucumber seedlings not only achieved a 56% control effect against cucumber gray mold, but also significantly increased the activity of various defense enzymes in the leaves, suggesting that *T. harzianum* mediates the activation of stress-defensing enzymes in the leaves to resist the attack of the pathogen (Huang et al., 2013). We investigated the inducing effect of T891 on the systemic resistance (ISR) in red kidney bean plants. It was found that SOD did not change significantly when *T. harzianum* T891 alone was applied compared to CK (*p* > 0.05), but SOD activity was remarkably higher under the stress of pathogenic fungus Fu13 in the treatments of (Fu13 and T891 + Fu13) (*p* < 0.05). SOD is an enzyme that scavenges free radicals of superoxide anion, which are thought to participate in most and senescence of plants and animals, therefore the magnitude of SOD activity can be used to evaluate the strength of plant disease resistance (Montalbini and Buonaurio, 1986). The elevation of SOD activity in red kidney bean treated with T891 indicated the activation of antioxidative enzyme SOD in defensing of the Fu13-induced oxidative stress. POD is a highly active enzyme in plants, which is not only involved in the polymerization process of lignin, but also an important scavenger of endogenous reactive oxygen species in the cell, which is actively involved in plant disease resistance (Joseph et al., 1998). POD were significantly increased in the groups of T891, Fu13 and the highest POD activity was recorded in T891 + Fu13 compared with CK (*p* < 0.05). This suggest that POD activity in plants can be increased by either *T. harzianum* or the pathogen, and the disease resistance can be improved through the joint action of the plant and *T. harzianum*. CAT activity is related to the metabolic strength of the plant as well as cold and disease resistance. Elevated CAT implies the alleviation of reactive oxygen species (ROS) damage to the plant cells (Legendre et al., 1993). Here, CAT activity was significantly increased in the T891 and T891 + Fu13 treatments as compared to CK (*p* < 0.05), suggesting that *T. harzianum* can improve the pathogen resistance of red kidney bean by increasing CAT activity, which is capable of scavenging excessive ROS. As a product of lipid peroxidation of cell membrane, MDA directly reflects the degree of membrane damage (Zhou et al., 2021). In this work, the MDA content in the treatments followed a trend of Fu13 > T891 + Fu13 > CK > T891, indicating that *T. harzianum* could alleviated membrane damage caused by the pathogen. In conclusion, the use of *T. harzianum* T891 enhanced the activities of induced systemic resistance (ISR) related enzymes POD and CAT and decrease the MDA content of red kidney bean plants (Figure 2), which helped better prevent and control of red kidney bean root rot, thereby promoted the growth of red kidney beans.

### 4.2 Effect of different treatments on the microbial community structure in the rhizosphere

Microorganisms are a crucial indicator for soil health (Yuan et al., 2020), and rhizosphere microbial communities are also considered critical for improving crop productivity, warding off soil-borne diseases, and maintaining the long-term health and function of the soil ecosystem (Trivedi et al., 2020). In the present study, there were no significant differences in the alpha diversity indices of bacterial and fungal communities between the T891 treatment and CK. This is similar to how *Trichoderma* affects the alpha diversity indices of rhizosphere microbial communities of soybean (*Glycine max*) reported by Labanya et al. (2018). The amount of *Trichoderma* applied may have an impact on these results (Liu and Hsiang, 1994). The alpha diversity indices of both bacterial and fungal communities were significantly lower in Fu13 treatment compared to the other three treatments, suggesting that the occurrence of root rot disease in the Fu13 treatment may be connected to the decrease in microbial alpha diversity. Previous studies have also found lower alpha diversity in the root systems of pathogen-infected plants relative to healthy plants (Lemanczyk and Sadowski, 2002). However, the reduction was reversed in Fu13 + T891 and the microbial alpha diversity was significantly higher (*p* < 0.05) compared to Fu13, indicating T891 may have played an important role reducing the incidence of root rot in red kidney bean by increasing the microbial alpha diversity in rhizosphere soil.

According to earlier research, disparities in microbial community composition may affect disease suppression capacity of soil and plant growth (Raaijmakers and Mazzola, 2016; Jin et al., 2017). Based on principal coordinate analysis (PCoA) and LEfSe analysis, it was found that exogenous microorganisms had a great impact on the structure of the microbiota. These changes in composition in terms of relative abundance can be ascribed to the invasion of *F. oxysporum* which enabled members of *Proteobacteria* and *Bacteroidota* to dominate in the soil environment, and led to the sharp decrease or increase in the specific phyla of bacteria or fungi in the other treatments, and thus changed the assembly of the soil microbial community.

Furthermore, we further find that there were marked disparities in the ASVs composition of bacteria and fungi among different treatments, and the bacteria may have a greater effect of reducing disease index and promoting plant growth in red kidney bean than fungi (Figure 6). Bacteria that varied significantly among different treatments may be closely associated with red kidney bean root rot and plant growth. The results revealed that 10 of the high abundance core ASVs were negatively correlated with both disease incidence and positively and correlated with plant biomass simultaneously. Whereas the relative abundance of *Porphyrobacter* (ASV 46), *Lysobacter* (ASV 85), *Microbacteriaceae* (ASV 105), and *Gemmatimonas* (ASV 107) was significantly (*p* < 0.05) higher in T891 + Fu13 compared to Fu13. *Porphyrobacter* has been reported to be a dominant group of bacteria with degrading ability (Hiraishi et al., 2002), *Lysobacter* is a biocontrol bacterium with great potential (Islam, 2010; Zhao et al., 2017), *Microbacteriaceae* have been shown to promote weight gain in *Salvia miltiorrhiza* hairy roots (You et al., 2018), *Gemmatimonas* can accelerate the nitrogen cycle and aggregate phosphate, thereby promoting plant growth (Zhang et al., 2023b). Randomized forest results also showed that *Porphyrobacter* (ASV 46) and *Lysobacter* (ASV 85) were the primary influencing factors (*p* < 0.05) on plant disease incidence and growth, respectively. Therefore, the restoration or enhancement of potentially beneficial host-associated bacterial microbiota induced by *T. harzianum* may be an effective means of controlling plant diseases and promoting plant biomass.

### 4.3 Effect of different treatments on the co-occurrence network of microbial communities

Microbial-microbial associations are critical for the functioning of micro-ecosystems in soils (Barberán et al., 2012). Molecular ecological networks (MENs) analysis can provide new insights into possible microbial correlations in diverse ecosystems (de Menezes et al., 2014). We used this method to construct and analyze interactions between microorganisms in rhizosphere soil after treatment of *T. harzianum* T891 or *F. oxysporum* Fu13, or both. The results showed that microbial associations in soils treated with *F. oxysporum* were more complex than those of other three groups (Figure 5). This aligns with the study of Tan et al. (2021). Root zone soil infected with *Ralstonia solanacearum* had a more complex network structure than the corresponding healthy samples. In addition, the corresponding topologies showed significant differences (Table 2). Specifically speaking, *F. oxysporum* Fu13-infected rhizospheric soils had the greatest amount of connections and the greatest average number of connections for each microbe, indicating that there were closer microbial associations within the network, which further strengthens the linkages between species in the community. However, the microbial network of rhizosphere soils in the T891 treatment had a higher ratio of positive/negative linkages, suggesting that the reshaped microbial community increased co-operation rather than competition, while the opposite was true for the Fu13 treatment. Hence we speculate it may be competition for nutrient elements or ecological niche space led to more diverse associations among microorganisms (Ghoul and Mitri, 2016), or that the disruption of microbial equilibrium by pathogen invasion led to stronger antagonistic interactions between fungal and bacterial members and pathogens (Garcia-Bayona and Comstock, 2018; Hu et al., 2020). In addition, large average path lengths indicate slow responses and high resistance of microbes to disturbance when perturbed by environmental changes (de Vries et al., 2018), and high clustering coefficients and modular community associations are favorable to the regulation of community stability (Eisenhauer et al., 2013; Downing et al., 2014), and thus are conducive to the control of pathogen reproduction and colonization. Compared to other groups, the microbial network of rhizosphere soils in T891 + Fu13 had higher mean path length, clustering coefficient, and modularization, suggesting a higher community stability formed after interactions and re-equilibrium among the inoculants and the indigenous members of the microbiota. In co-occurrence network analyses, key nodes are generally considered to play a non-negligible impact in maintaining the stability of microbial ecosystems (Fan et al., 2018). Through our MENs analysis, 10 ASVs were found in Fu13 and 19 ASVs were found in T891 + Fu13 as key nodes, and in comparison to the other three groups, the Fu13 treatment had a significantly lower abundance of key node ASVs (*p <* 0.05), which may be effective in improving the resistance of red kidney bean against the pathogen (Figure 5). Also, the differences in key ASVs in different groups demonstrated that different treatments significantly affected the community diversity and structure of soil microorganisms.

## 5 Conclusion

In summary, we explored the effects of pre-inoculation of T891 on root rot control, plant growth, plant defense enzyme activities, and rhizosphere microbial community diversity and structure in red kidney bean subjected to infestation of Fu13, the causal agent of red kidney bean root rot. Pre-inoculation with T891 significantly lowered the incidence and severity of red kidney bean root rot induced by Fu13, improved plant growth indexes, and upregulated activity of defense enzymes CAT and POD in the systemic resistance of red kidney bean. These effects were consistent with significant alterations of rhizosphere soil microbial community, most notably a significant decline in plant pathogens. Furthermore, the application of T891 induced the recovery or enhancement of potentially beneficial host-associated bacterial microorganisms, resulting in a more stable structure of the microbial network, which is an effective means of controlling plant diseases and increasing plant biomass in red kidney bean. In conclusion, *T. harzianum* is effective in the control of red kidney bean root rot by improving the defense enzyme activity in kidney bean, recruiting beneficial rhizospheric microorganisms and reshaping the soil microbial community.

## Data availability statement

The datasets presented in this study can be found in online repositories. The names of the repository/repositories and accession number(s) can be found at: https://www.ncbi.nlm.nih.gov/, PRJNA1047896.

## Author contributions

ZG: Writing – original draft, Writing – review & editing. JZ: Writing – original draft, Writing – review & editing. ZL: Writing – original draft. YLi: Writing – original draft. ML: Writing – original draft. QM: Writing – original draft, Writing – review & editing. ZY: Writing – review & editing. YLu: Writing – original draft. QZ: Writing – original draft, Writing – review & editing. MY: Writing – original draft, Writing – review & editing.
