# Supplementary material for: Trichoderma harzianum prevents red kidney bean root rot by increasing plant antioxidant enzyme activity and regulating the rhizosphere microbial community
Source: Front Microbiol. 2024 Mar 20;15:1348680. doi: 10.3389/fmicb.2024.1348680 (PMC10987954; doi:10.3389/fmicb.2024.1348680)
Supplement: Supplementary file 1 [file Table_1.docx]

Supplementary Material

*Trichoderma harzianum* prevents red kidney bean root rot by increasing plant antioxidant enzyme activity and regulating the rhizosphere microbial community

Zhifen Guo, Jiaxing Zhang, Zhibin Liu, Yu Li, Meng Li, Qiuxia Meng, Zhiping Yang, Yuan Luo, Qiang Zhang* and Min Yan*

*** Correspondence:**

Qiang Zhang: [zhangqiang0351@163.com](mailto:zhangqiang0351@163.com)

Min Yan: ymrice@163.com

**List Supplementary Materials:**

**Supplementary Figure S1.** Bacterial and fungal community diversity (Shannon) (A) and evenness (Pielou_e) (B) for different treatments. Box plots show the first and third quartiles with horizontal bars at the median and whisker lines show the range of outliers, not exceeding 1.5 times the interquartile range. **p*<0.05, ***p*<0.01, ****p*<0.001 according to Tukey’s test.

**Supplementary Figure S2.** Linear discriminant analysis (LDA) effect sizes (LEFSe) for bacterial taxa (A) and fungal taxa (B) identifying taxa that were most differentially enriched across treatments. Different colored circles represent taxa that were significantly enriched across treatments (LDA>4.5, Wilcoxon test, *p*<0.05).

**Supplementary Table S1.** Differential enrichment of ASVs across treatments compared to CK.

# Supplementary Figures and Tables

## Supplementary Figures


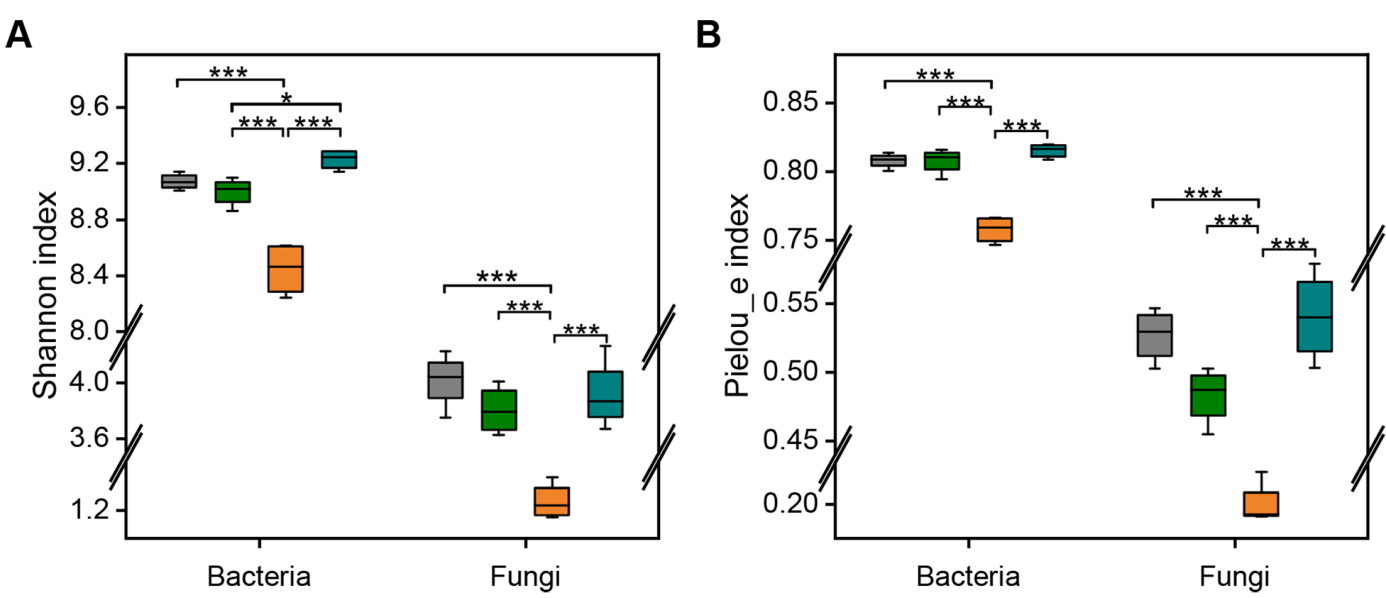


**Supplementary Figure S1.** Bacterial and fungal community diversity (Shannon) (A) and evenness (Pielou_e) (B) for different treatments. Box plots show the first and third quartiles with horizontal bars at the median and whisker lines show the range of outliers, not exceeding 1.5 times the interquartile range. **p*<0.05, ***p*<0.01, ****p*<0.001 according to Tukey’s test.


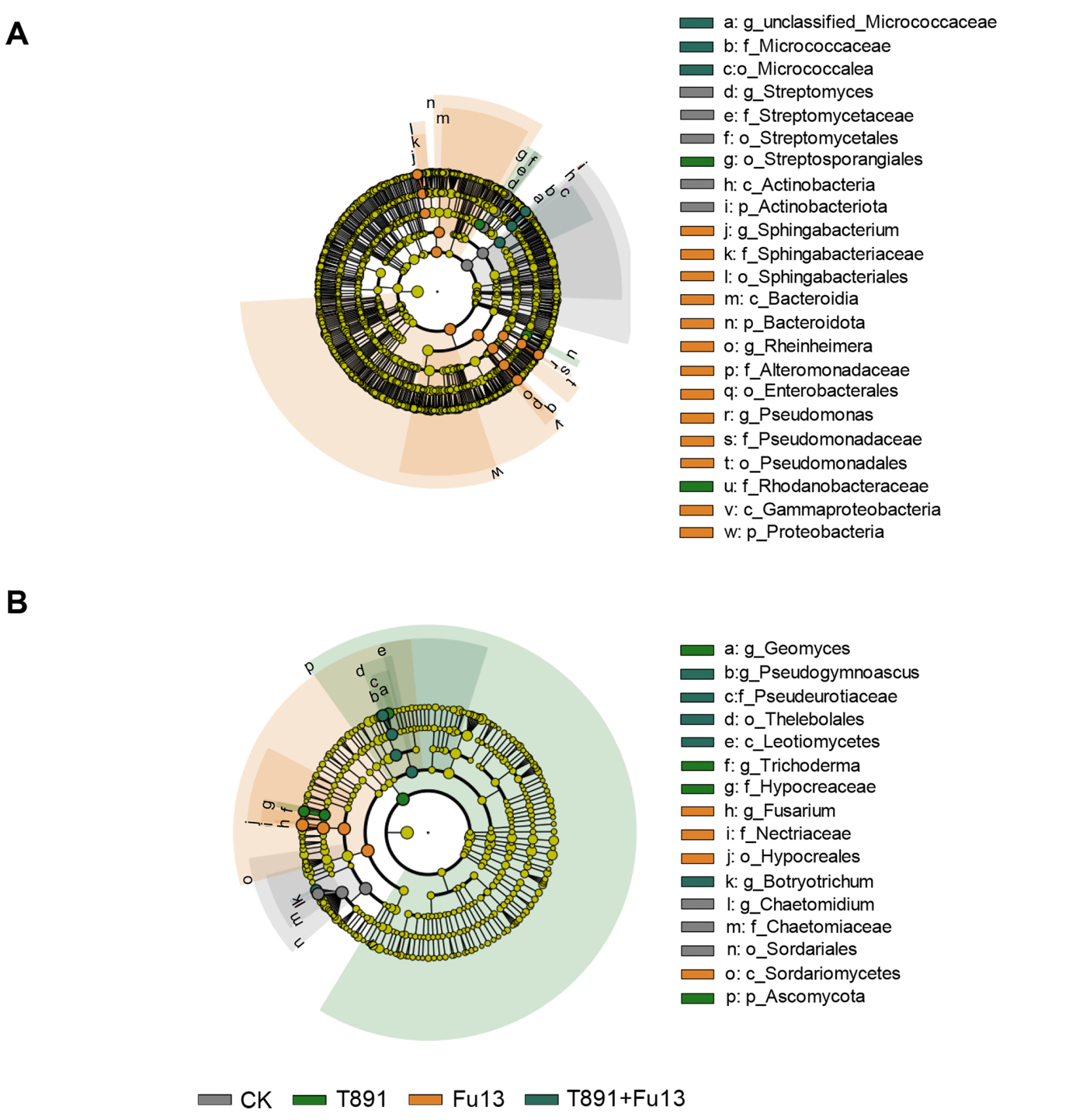


**Supplementary Figure S2.** Linear discriminant analysis (LDA) effect sizes (LEFSe) for bacterial taxa (A) and fungal taxa (B) identifying taxa that were most differentially enriched across treatments. Different colored circles represent taxa that were significantly enriched across treatments (LDA>4.5, Wilcoxon test, *p*<0.05).

## Supplementary Tables

**Supplementary Table S1.** Differential enrichment of ASVs across treatments compared to CK.

|  | Treat | ID | log2F | padj |  | ID | log2F | padj |
| --- | --- | --- | --- | --- | --- | --- | --- | --- |
| bacteria | T891 | ASV 32 | 3.36 | 2.85E-11 | fungi | ASV 4 | 11.00 | 3.97E-17 |
|  |  | ASV 133 | 2.37 | 0.004183 |  |  |  |  |
|  |  | ASV 162 | -2.09 | 6.10E-23 |  |  |  |  |
|  | Fu13 | ASV 1 | -2.22 | 2.13E-47 |  | ASV 1 | 14.66 | 0 |
|  |  | ASV 9 | -2.53 | 6.44E-39 |  | ASV 2 | 2.31 | 5.77E-12 |
|  |  | ASV 15 | -3.00 | 2.50E-40 |  | ASV 18 | -2.34 | 0.013772 |
|  |  | ASV18 | 2.48 | 6.31E-24 |  |  |  |  |
|  |  | ASV 17 | -4.59 | 2.62E-05 |  |  |  |  |
|  |  | ASV 26 | 8.11 | 5.55E-15 |  |  |  |  |
|  |  | ASV 32 | 8.30 | 2.92E-36 |  |  |  |  |
|  |  | ASV 23 | -4.37 | 1.01E-13 |  |  |  |  |
|  |  | ASV 41 | -3.37 | 3.32E-08 |  |  |  |  |
|  |  | ASV 46 | -2.96 | 0.005952 |  |  |  |  |
|  |  | ASV 62 | 2.94 | 2.72E-19 |  |  |  |  |
|  |  | ASV 66 | 4.77 | 1.29E-53 |  |  |  |  |
|  |  | ASV 70 | 4.02 | 3.38E-28 |  |  |  |  |
|  |  | ASV 68 | -3.06 | 1.99E-18 |  |  |  |  |
|  |  | ASV 77 | 5.54 | 3.21E-56 |  |  |  |  |
|  |  | ASV 81 | 3.14 | 1.68E-20 |  |  |  |  |
|  |  | ASV 71 | -3.24 | 9.01E-11 |  |  |  |  |
|  |  | ASV 88 | 3.68 | 3.50E-07 |  |  |  |  |
|  |  | ASV 80 | 2.04 | 1.84E-24 |  |  |  |  |
|  |  | ASV 85 | -2.15 | 0.001107 |  |  |  |  |
|  |  | ASV 99 | 2.25 | 2.72E-19 |  |  |  |  |
|  |  | ASV 118 | 2.73 | 9.18E-17 |  |  |  |  |
|  |  | ASV 105 | -3.29 | 9.71E-05 |  |  |  |  |
|  |  | ASV 100 | -2.18 | 0.004374 |  |  |  |  |
|  |  | ASV 107 | -3.26 | 3.33E-05 |  |  |  |  |
|  |  | ASV 137 | 3.38 | 2.43E-15 |  |  |  |  |
|  |  | ASV 128 | -2.69 | 0.001227 |  |  |  |  |
|  |  | ASV 133 | 6.73 | 4.77E-27 |  |  |  |  |
|  |  | ASV 151 | 2.71 | 9.99E-24 |  |  |  |  |
|  |  | ASV 166 | 4.08 | 1.12E-21 |  |  |  |  |
|  |  | ASV 162 | -2.36 | 2.71E-07 |  |  |  |  |
|  |  | ASV 178 | 3.88 | 1.80E-22 |  |  |  |  |
|  |  | ASV 168 | -2.32 | 2.20E-05 |  |  |  |  |
|  | T891+Fu13 | ASV 1 | -2.88 | 2.67E-82 |  | ASV 1 | 11.24 | 0 |
|  |  | ASV 9 | -2.76 | 2.01E-76 |  | ASV 2 | -2.40 | 8.70E-30 |
|  |  | ASV 15 | -3.88 | 4.92E-90 |  | ASV 3 | 4.22 | 1.94E-28 |
|  |  | ASV 18 | 2.079 | 1.37E-12 |  | ASV 4 | 9.51 | 4.05E-96 |
|  |  | ASV 17 | -3.11 | 2.11E-99 |  | ASV 6 | -2.91 | 8.91E-20 |
|  |  | ASV 26 | 3.59 | 0.004910 |  | ASV 10 | 3.27 | 8.70E-30 |
|  |  | ASV 32 | 3.38 | 5.27E-09 |  | ASV 18 | 4.01 | 6.37E-23 |
|  |  | ASV 23 | -4.58 | 8.81E-54 |  | ASV 17 | 3.04 | 5.02E-20 |
|  |  | ASV 41 | -2.71 | 7.68E-13 |  | ASV 22 | 3.30 | 8.50E-34 |
|  |  | ASV 62 | 2.22 | 1.23E-19 |  | ASV 20 | 2.39 | 1.84E-18 |
|  |  | ASV 81 | 3.03 | 2.10E-18 |  |  |  |  |
|  |  | ASV 71 | -3.00 | 3.90E-15 |  |  |  |  |
|  |  | ASV 88 | 3.56 | 3.56E-06 |  |  |  |  |
|  |  | ASV 97 | -3.09 | 0.021165 |  |  |  |  |
|  |  | ASV 99 | 2.22 | 4.14E-17 |  |  |  |  |
|  |  | ASV 118 | 3.43 | 6.73E-29 |  |  |  |  |
|  |  | ASV 110 | 2.04 | 8.10E-11 |  |  |  |  |
|  |  | ASV 109 | -2.57 | 3.64E-17 |  |  |  |  |
|  |  | ASV 137 | 3.39 | 5.25E-19 |  |  |  |  |
|  |  | ASV 133 | 6.49 | 4.23E-27 |  |  |  |  |
|  |  | ASV 143 | 2.32 | 7.31E-17 |  |  |  |  |
|  |  | ASV 150 | 2.05 | 4.44E-08 |  |  |  |  |
|  |  | ASV 162 | -2.89 | 5.63E-22 |  |  |  |  |
|  |  | ASV 178 | 2.12 | 9.75E-08 |  |  |  |  |
